# Assessment of retrospective collection of EQ-5D-5L in a US COVID-19 population

**DOI:** 10.1186/s12955-023-02187-x

**Published:** 2023-09-08

**Authors:** Xiaowu Sun, Manuela Di Fusco, Laura Puzniak, Henriette Coetzer, Joann M. Zamparo, Ying P. Tabak, Joseph C. Cappelleri

**Affiliations:** 1 Clinical Trial Services, CVS Health, Woonsocket, RI USA; 2 Pfizer Inc, Health Economics and Outcomes Research, New York, NY USA; 3 Pfizer Inc, MDSCA Vaccines, Collegeville, PA USA; 4 Pfizer Inc, Groton, CT USA; 5 Pfizer Inc, Statistical Research and Data Science Center, Groton, CT USA

**Keywords:** COVID-19, SARS-CoV-2, Health-related quality of life, Retrospective collection

## Abstract

**Background:**

It is imperative to evaluate health related quality of life (HRQoL) pre-COVID-19, but there is currently no evidence of the retrospective application of the EuroQol 5-Dimension, 5 level version (EQ-5D-5L) for COVID-19 studies.

**Methods:**

Symptomatic patients with SARS-CoV-2 at CVS Health US test sites were recruited between 01/31/2022-04/30/2022. Consented participants completed the EQ-5D-5L questionnaire twice: a modified version where all the questions were past tense to retrospectively assess pre-COVID-19 baseline QoL, and the standard version in present tense to assess current HRQoL. Duncan’s new multiple range test was adopted for post analysis of variance pairwise comparisons of EQ visual analog scale (EQ VAS) means between problem levels for each of 5 domains. A linear mixed model was applied to check whether the relationship between EQ VAS and utility index (UI) was consistent pre-COVID-19 and during COVID-19. Matching-adjusted indirect comparison was used to compare pre-COVID-19 UI and EQ VAS scores with those of the US population. Lastly, Cohen’s d was used to quantify the magnitude of difference in means between two groups.

**Results:**

Of 676 participants, 10.2% were age 65 or more years old, 73.2% female and 71.9% white. Diabetes was reported by 4.7% participants and hypertension by 11.2%. The estimated coefficient for the interaction of UI-by-retrospective collection indicator (0 = standard prospective collection, 1 = retrospective for pre-COVID-19), -4.2 (SE: 3.2), P = 0.197, indicates that retrospective collection does not significantly alter the relationship between EQ VAS and UI. After adjusting for age, gender, diabetes, hypertension, and percent of mobility problems, the predicted means of pre-COVID-19 baseline EQ VAS and UI were 84.6 and 0.866, respectively. Both means were close to published US population norms (80.4 and 0.851) compared to those observed (87.4 and 0.924). After adjusting for age, gender, diabetes, and hypertension, the calculated ES between pre-COVID-19 and COVID-19 for UI and EQ VAS were 0.15 and 0.39, respectively. Without retrospectively collected EQ-5D-5L, using US population norms tended to underestimate the impact of COVID-19 on HRQoL.

**Conclusion:**

At a group level the retrospectively collected pre-COVID-19 EQ-5D-5L is adequate and makes it possible to directly evaluate the impact of COVID-19 on HRQoL. (ClinicalTrials.gov NCT05160636)

**Supplementary Information:**

The online version contains supplementary material available at 10.1186/s12955-023-02187-x.

## Background

The impact of COVID-19 extends beyond clinical outcomes. To holistically understand the burden of COVID-19, it is important to measure its impact on patients’ quality of life. The EuroQoL Group 5 dimension and 5 levels (EQ-5D-5L) instrument [1] is an internationally validated questionnaire that is widely used for measuring health-related quality of life (HRQoL) and deriving utilities for estimation of Quality Adjusted Life Years (QALYs). Retrospective utilization of the EQ-5D-5L to assess pre-event status is limited, but the feasibility and validity of its retrospective collection (EQ-5D-5L) to assess past HRQoL was investigated in prior studies [2, 3]. In Lawson et al. (2020) [2], EQ-5D-5L was collected prospectively in patients 2 weeks prior to their date of elective hip or knee arthroplasty surgery and then retrospectively collected following their operation to recall their pre-operative health status. At a group level the agreement was high between prospective and retrospective measurements indicating retrospective collection could be valid in orthopedic clinical context.

Another study by Rajan et al. (2021) [3] utilized the EQ-5D-5L prospectively among patients with a stroke at 3 months post-discharge and compared those results with the retrospective collection of their 3rd month EQ-5D-5L at 6th, 9th, or 12th month after hospital discharge. Considerable agreement was observed in mobility, self-care, and usual activities dimensions of EQ-5D-5L based on weighted kappa (range 0.72–0.95), and concordance was good to excellent for utility score based on intraclass correlation coefficients (range 0.79–0.81). The authors concluded that retrospective collection of EQ-5D-5L could be a valid alternative for assessing morbid health status.

Retrospective and prospective collection of EQ-5D-5L have been used in infectious disease PRO studies to establish the baseline pre-infection health status and the morbid health status over the course of the infection [4, 5]. However, the validity of retrospective collection of EQ-5D-5L was not assessed in those studies. A recent study utilizing retrospective and prospective collection of EQ-5D-5L was used in a PRO study of US symptomatic outpatients with positive reverse transcription–polymerase chain reaction for SARS-CoV-2. EQ-5D-5L was used to establish, respectively, the baseline pre-COVID-19 health status and the health status at week 1 and week 4 following infection. Subsequently, the pre-infection and COVID-19 health statuses were compared to evaluate the impact of COVID-19 on HRQoL [6]. This subsequent methodology study utilizing the PRO data collected in the COVID-19 study tests the validity of the use of retrospective collection of EQ-5D-5L for establishing pre-infection status for the first time for COVID-19-related studies.

## Methods

### Data source

In the COVID-19 PRO study [6], participants were recruited between 01/31/2022 and 04/30/2022 among US adult outpatients with ≥ 1 self-reported symptom and positive reverse transcription–polymerase chain reaction test for SARS-CoV-2 at CVS Health test sites (ClinicalTrials.gov NCT05160636). The analytic population for the Di Fusco et al. study was limited to participants unvaccinated or receiving the BNT162b2 COVID-19 vaccine only. To assess the validity of the retrospective collection methodology, all patients that were enrolled were included [6].

### EQ-5D-5L

EQ-5D-5L addresses quality of life across five dimensions (mobility, self-care, usual activities, pain/discomfort, and anxiety/depression) and five levels (no problems, slight problems, moderate problems, severe problems, extreme problems/unable). These five domains were converted into the Utility Index (UI) using the US-based weights [7]. Moreover, it addresses a general assessment of health, the EQ visual analog scale (EQ VAS), via a 101-point Visual Analog Scale (EQ VAS) ranging from 0 = “Worst imaginable health state” to 100 = “Best imaginable health state” [1]. On the day of enrollment (~ day 3 after testing positive to SARS-CoV-2) consented participants completed the EQ-5D-5L questionnaire twice a modified version where all the questions were past tense to retrospectively assess pre-COVID-19 baseline HRQoL and, separately, the standard version in present tense to assess current HRQoL. These two versions were administered consecutively with no washout or break between, and in random order to balance the potential responder bias due to the order of administration.

### Statistical methods

To support the validity of retrospectively collected EQ-5D-5L, the following assumptions were utilized: firstly, lower EQ VAS is associated with higher problem levels within each dimension of EQ-5D-5L. Secondly, our pre-COVID-19 cohort can be considered as a sample from the general US population. After adjusting for major distributional differences, mean EQ VAS should be close to the population norm. Thirdly, retrospective collection does not modify the relationship between EQ VAS and UI. Last, retrospectively collected EQ VAS is more appropriate than the population norm when evaluating COVID-19’s impact on HRQoL.

Categorical variables were described by using frequency and percentages. Continuous variables were described by using means and standard deviations. The analysis of variance (ANOVA) was used to test differences in means between different problem levels within each domain of EQ-5D-5L. Duncan’s new multiple range test was adopted for pairwise comparisons post ANOVA [8, 9], which may evaluate the association between EQ VAS and problem levels.

A linear mixed model was used to characterize the relationship between EQ VAS and UI [10]. The response variable was EQ VAS. The explanatory variables included UI, the indicator for retrospective assessment variable RETRO (1 = retrospective collection for pre-COVID-19, 0 = standard collection for day 3 after COVID-19 testing) and its interaction with UI. The coefficient of the interaction term reflects the magnitude of relationship altered by retrospective assessment between EQ VAS and UI. Random intercepts were incorporated to account for the clusters of assessments within participants.

Matching-adjusted indirect comparison (MAIC) [11] was used to compare pre-COVID-19 UI and EQ VAS scores with those of the US population. To do that, weights for individuals in our sample were estimated so that weighted percentage of selected patient characteristics matched those published. Then two-sample t-tests were used to test differences between weighted EQ VAS or UI with those in the US population, as reported in the Jiang et al. (2021) face-to-face sample [12].

The effect size (ES), Cohen’s d, was calculated to assess the magnitude of difference in means between two groups [13, 14]. Specifically, when comparing COVID-19 to pre-COVID-19 baseline, the ES was calculated as mean score differences divided by the standard deviation of score difference. When comparing the COVID-19 to US norm, the ES was calculated as the difference in mean COVID-19 score and US norm, divided by the pooled standard deviation of scores. Values of 0.2, 0.5, and 0.8 standard deviation (SD) units represent small, medium, and large ES, respectively. These cut-off estimates have been widely used to establish important differences in HRQoL studies [15].

Among 676 participants submitting responses to the EQ-5D-5L questionnaire, missingness was recorded in 7 instances only: 2 participants did not answer the EQ VAS component in the EQ-5D-5L retrospective version and 5 participants missed the EQ VAS component in the standard version. There were no missing data for utility index scores. The impact of missing data was considered minimum, so the analyses were all based on available data.

## Results

Of 676 participants, 10.2% were age 65 or older, 73.2% female and 71.9% white. Asthma or chronic lung disease, diabetes, and hypertension were reported by 8.6%, 4.7%, and 11.2% participants, respectively. (Table 1) Descriptive statistics are presented in Supplemental Table 1.

**Table 1:** Retrospective Collection of EQ-5D-5L Utility Index (US Preference Weights) and EQ VAS by Respondent Characteristics

	Index Score		EQ VAS	
	n (%)	Mean (SD)	n (%)	Mean (SD)
All	676	0.924 (0.117)	674	87.4 (10.9)
Age				
18–34	236 (34.9)	0.916 (0.111)	236 (35.0)	88.4 (9.2)
35–64	371 (54.9)	0.927 (0.122)	370 (54.9)	86.5 (12.1)
65+	69 (10.2)	0.936 (0.106)	68 (10.1)	88.8 (9.2)
Gender				
Female	495 (73.2)	0.920 (0.113)	495 (73.4)	87.2 (11.1)
Male	181 (26.8)	0.935 (0.125)	179 (26.6)	87.9 (10.3)
Race/Ethnicity				
White or Caucasian	486 (71.9)	0.921 (0.118)	484 (71.8)	87.1 (10.7)
Black or African American	32 (4.7)	0.918 (0.166)	32 (4.8)	82.7 (14.8)
Hispanic	85 (12.6)	0.930 (0.105)	85 (12.6)	88.4 (11.4)
Asian	35 (5.2)	0.969 (0.051)	35 (5.2)	89.9 (9.0)
Patient Refused	16 (2.4)	0.927 (0.096)	16 (2.4)	86.6 (10.2)
Other	22 (3.3)	0.899 (0.131)	22 (3.3)	91.7 (9.1)
Chronic conditions				
Asthma or Chronic Lung Disease	58 (8.6)	0.873 (0.152)	57 (8.5)	81.7 (14.0)
Diabetes	32 (4.7)	0.887 (0.131)	32 (4.8)	82.8 (11.1)
Hypertension	76 (11.2)	0.887 (0.158)	75 (11.1)	83.1 (12.2)
Extreme obesity	23 (3.4)	0.782 (0.258)	23 (3.4)	77.0 (13.7)
Immunocompromised Conditions or Weakened Immune System ^a^	27 (4.0)	0.889 (0.134)	27 (4.0)	78.6 (15.2)

^a^ Immunocompromised conditions includes compromised immune system (such as from immuno-compromising drugs, solid organ or blood stem cell transplant, HIV, or other conditions), conditions that result in a weakened immune system, including cancer treatment, and kidney failure or end stage renal disease

EQ VAS = EuroQol Visual Analogue Scale

**Fig. 1:**
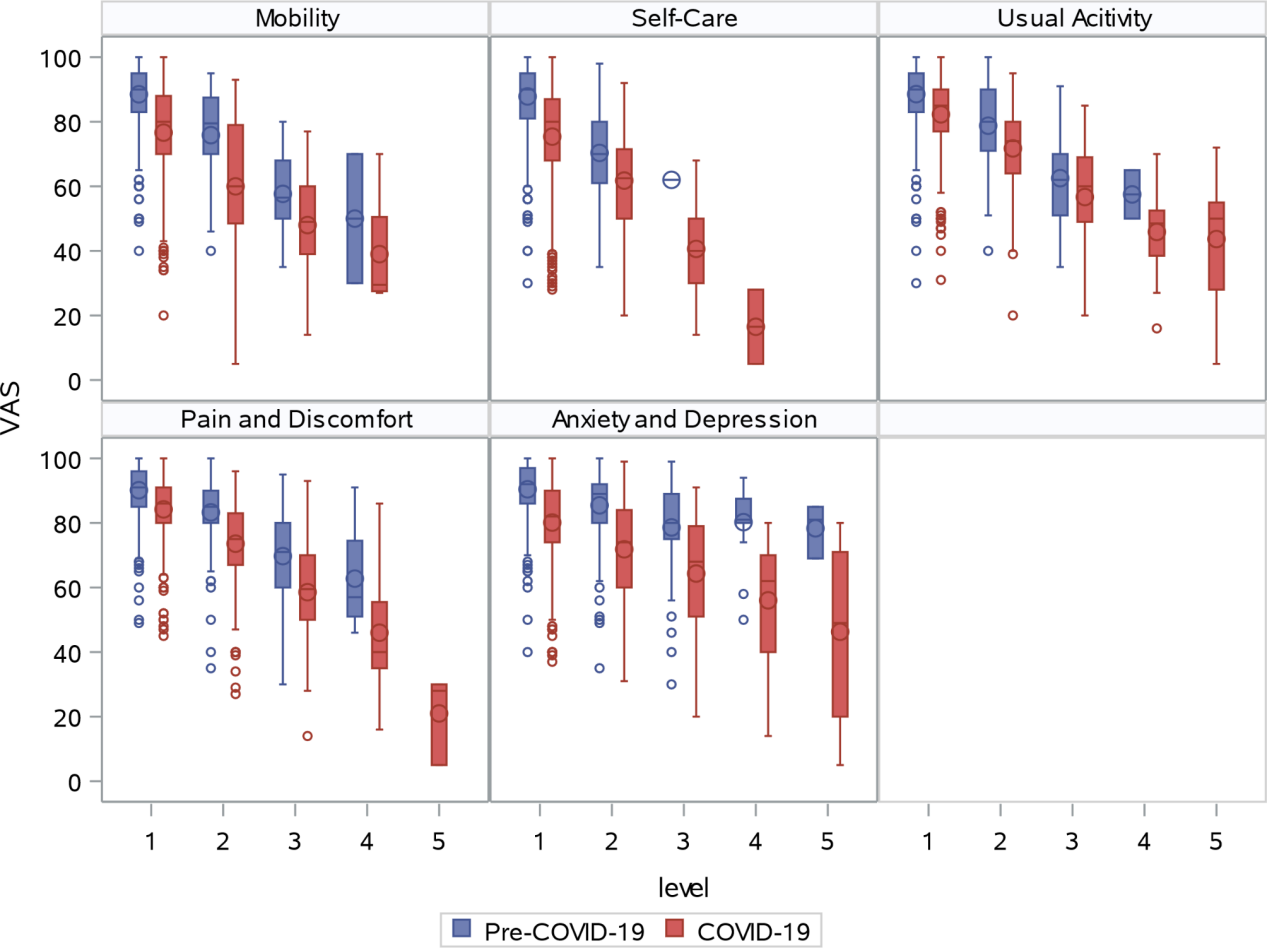
Sample Means of EQ VAS by EQ-5D-5L Domain Level: Pre-COVID-19 and COVID-19

As shown in Fig. 1, worse EQ VAS was associated with a higher level of severity in each EQ-5D-5L domain, for both retrospectively collected pre-COVID-19 baseline and standard collection for day 3 after COVID-19 positive testing. A detailed summary of EQ VAS by EQ-5D-5L domain level for retrospective collection for pre-COVID-19 and standard collection for COVID-19, as well as the pairwise comparisons between levels of each domain, are presented in Supplemental Table 2. For pre-COVID-19 mobility, mean EQ VAS scores were 88.5, 75.9, 57.7 and 50.0 for those reporting no problem, slight problem, moderate problem, and severe problem, respectively. No participants reported ‘unable’ for mobility. According to Duncan’s tests, mean EQ VAS was found to be significantly different among no problem, slight problem, and moderate/severe problem categories. In addition, a summary of EQ VAS by health states with a sample size greater than 10 is presented in Supplemental Table 3.

Compared with the US general population [12], the current cohort was predominantly female, white, and reported fewer chronic conditions. The pre-COVID-19 baseline mean utility index (UI) of 0.924 and mean EQ VAS of 87.4 were higher than those in the US population (0.851 and 80.4, respectively). In addition, for all five domains, less problems were reported, which were prevalent in 7.1%, 6.7%, 7.9%, 27.3% and 43.5% of the current study cohort versus 28.4%, 6.5%, 24.7%, 51.0%, and 38.4% in Jiang et al. (2021) [12] for mobility, self-care, usual activity, pain/discomfort and anxiety/depression, respectively.

For the model predicting EQ VAS by using UI, RETRO and their interaction, the estimated coefficient of UI-by-RETRO interaction term was − 4.2 (SE: 3.2), P = 0.197, which indicated that the magnitude of relationship between EQ VAS and UI was not significantly altered by retrospective assessment.

To compare with the US population norms for the EQ-5D-5L [12], we matched percentages of age category, gender, diabetes, and hypertension in our sample to US population norms, age category (31.6% for 18–34, 48.7% for 35–64 and 19.7% for ≥ 65 years), gender (49.7% for male and 49.8 for female), diabetes (9.8%), and hypertension (23.8%), by using the MAIC approach [11]. After weighting, the effective sample size reduced to 440. The weighted mean of UI and EQ VAS for pre-COVID-19 baseline decreased slightly to 87.0 and 0.922, respectively. Both were significantly higher than US population norms, P < 0.001. When percent of problems with mobility was added, the effective sample size reduced to 291. The weighted mean of UI for pre-COVID-19 baseline became 0.866 (SD: 0.176), which is not statistically different from the US population norm 0.851 (P = 0.253). The weighted mean of EQ VAS was 84.6 (SD:12.8) and higher than the US population norm of 80.4 (P < 0.001). (Table 2)

**Table 2:** Observed and Weighted EQ-5D-5L Assessments with Comparisons with US Population Norms

	n	UI	EQ VAS	Percent with problem				
				MO	SC	UC	PD	AD
Pre-COVID-19								
Observed	676	0.924 (0.117)	87.4 (10.9)	7.3	2.7	7.6	27.8	43.5
MAIC 1	440 ^a^	0.922 (0.125)	87.0 (10.8)	8.9	3.1	8.2	27.6	41.0
MAIC 2	291 ^a^	0.866 (0.176)	84.6 (12.8)	28.4	9.0	16.6	40.9	47.4
COVID-19								
Observed	671	0.808 (0.204)	73.3 (16.9)	17.2	11.4	51.3	67.3	53.7
MAIC 1	440 ^a^	0.820 (0.192)	74.3 (16.8)	19.0	10.4	47.9	63.5	50.6
Jiang et al. (2021) (12)	1,134	0.851 (0.205)	80.4 (15.6)	28.4	6.5	24.7	51.0	38.4
Cha et al. (2019) (16) ^b^	1,047	Not available	84.6 (14.5)	25.2	6.9	21.8	40.1	29.6

^a^ effective sample size due to weighting

^b^ The sample of EQ-5D-3 L sub-study

UI = utility index; EQ VAS = EuroQol visual analog scale; MO = Mobility; SC = Self-care; UC = Usual activity; PD = Pain/Discomfort; AD = Anxiety/Depression

MAIC 1: Matching percentages of age (18–34,30–64 and 65+), gender, diabetes, and hypertension

MAIC 2: MAIC 1 plus percentage of problems (yes/no) with mobility

At approximately day 3 after COVID-19 positive testing, UI and EQ VAS were 0.808 and 73.3, respectively. When compared with baseline assessment, Cohen’s d for UI and EQ VAS were 0.68 and 1.01, a medium-to-large impact on UI and a large impact on EQ VAS, respectively. When compared with US population norms [12], Cohen’s d for UI and EQ VAS were 0.21 and 0.44, a small impact on UI and a small-to-medium impact on EQ VAS, respectively. However, the percentage of problems with mobility, 17.2%, of COVID-19 cohort was significantly lower than 25.2% of US population [12], P < 0.001. After matching on percentages of age category (18–29, 30–64 and ≥ 65 years), gender, diabetes, and hypertension, 19.0% with mobility problems was still significantly lower than 25.2%, P < 0.001. The calculated ES for UI and EQ VAS became 0.15 and 0.39, respectively. (Table 2)

## Discussion

In this current study implementing retrospective collection of EQ-5D-5L data, EQ VAS generally declined along with more severe levels in each of 5 domains as expected. Strong empirical evidence indicates that retrospective collection did not materially alter the relationship between EQ VAS and UI. Our current study population was predominantly female, white, and reported fewer impairments on the EQ VAS than the general US population. After adjusting for demographic variables, chronic conditions, and percentage of mobility problems, mean scores of EQ VAS and UI approached those of US population norms. As with related studies which demonstrate the validity of retrospective collection of EQ-5D-5L through direct comparisons with standard collection [2, 3], results from the current study provide evidence to support the validity of retrospective EQ-5D-5L collection when it is not feasible to collect this information prospectively. To our knowledge, however, this is the first study of its kind in an adult outpatient COVID-19 cohort.

The pre-COVID-19 adjusted mean EQ VAS of 84.6 is the same as that reported for the US general population in Cha et al. (2019), the 2017 sample of EQ-5D-3 L sub-study [16], but still higher than that reported by the face-to-face sample in Jiang et al. (2021) [12]. This difference may be due to under-adjustment for participants’ characteristics and possible bias in retrospective assessment. Another possibility is due to the nature of data collection in our study (online). Jiang et al. (2021) [12] reported a mean 3.0-point lower in online respondents than the face-to-face respondents. However, differences in administration mode cannot explain the higher adjusted value in our study.

While useful to adjust for differences in participants’ characteristics between study cohort and the cohort deriving US population norms, there are limitations with MAIC. In the current study chronic conditions were reported less than US general population and Jiang et al. (2021) [12]. Nevertheless, conditions that are either not commonly available in both studies or not consistently collected cannot be adjusted, which resulted in adjustment for diabetes and hypertension only. The addition of retrospectively collected problems with mobility for pre-COVID-19 in MAIC brought EQ VAS and UI much closer to US population norms.

When it is not feasible to collect prospective measurements on EQ-5D-5L, as well as EQ VAS, the current evidence suggests that it reasonable to consider retrospective assessment under the situation found in our study, including the short recall period (only a few days) for the retrospective assessment. Especially for mobility, there is high agreement between retrospective collection and standard collection [2, 3]. High percentage of no reported mobility problems is in alignment with an ambulatory cohort comprising of individuals able to walk into a store for COVID-19 testing. Even so, we did not attempt to use problem levels where there were either too few to be reliably adjusted or we were unable to adjust for the level not observed. (Supplemental Table 2)

The retrospective assessment of EQ VAS for pre-COVID-19 may either be recalled directly, or can be reconstructed based on the participant’s current state of COVID-19 and the assumptions of change from pre-COVID-19 to COVID-19. The recall bias occurs because of errors and distortions in the recollection of pre-COVID-19 state as well as in the inferential process from pre-COVID-19 to COVID-19 [17]. As shown in Rajan et al. (2021) [3], stroke patients tended to underestimate EQ VAS retrospectively, which supports the ‘present state effect’ [17, 18], that is, a person who feels well might think his status improved and therefore tends to underestimate his previous state. Conversely, a patient with COVID-19 who feels bad might think that her status worsened and therefore tends to overestimate her pre-COVID-19 status. As presented in Supplemental Tables 1, the mean EQ VAS score was 85.3 if administered first for pre-COVID-19, and 89.7 if after standard version.

Had EQ-5D-5L not been collected retrospectively, US population norms must be compared to evaluate COVID-19’s impact on HRQoL. The assumption is that COVID-19 causes more problems in mobility, self-care, usual activity, pain/discomfort and anxiety/depression, as well as overall health. However, our current cohort of patients with COVID-19 had less problems with mobility than the US general population, whether matching on a selected patient’s characteristics or not. On the other hand, retrospectively collected baseline percentage of problems with anxiety and depression, 43.5%, was higher than US population norm, 38.4%, based on the cohort pre-pandemic [12], which is consistent with World Health Organization report about elevated prevalence of anxiety and depression during COVID-19 pandemic [19]. Comparisons of UI and EQ VAS of patients with COVID-19 with population norms cannot account for such differences, which leads to unreliable estimates of COVID-19’s impact. Last, population norms may not be available in the general population. Even for EQ-5D-5L, the norms were developed based on a quota-sampling of the US population, which may not provide a true norm as larger random samples can provide.

Of 39,889 candidates with email invitations, 676 patients consented to participate and completed online surveys. Comparisons between the study cohort and those not included in the study were presented in Supplemental Table 4. Patients with COVID-19 vaccination, 30 years or older, female, white and living in less vulnerable communities were more likely to respond to the study. Study participants were more likely to have one or more comorbidities but had similar percentages of acute COVID-19 symptoms as those not included in the study. Though nonresponse bias is a potential limitation in this study, researchers have shown little relationship between survey response rate and nonresponse bias [20, 21]; nevertheless, that may not be generalizable to self-reported HRQoL in the current setting.

In summary, limitations in this study include: the lack of a “true” comparator for pre COVID-19 HRQoL because one does not exist as of yet, potential bias in retrospective assessment, and unknown sources of nonresponse bias. Further research is needed to directly compare standard prospective assessment of pre-COVID-19 EQ-5D-5L with retrospective assessment. Results in this analysis should therefore be interpreted with caution.

## Conclusions

At a group level, the retrospectively collected pre-COVID-19 EQ-5D-5L is adequate when compared with the US population norm and reasonably aligned when compared with standard collection of EQ-5D-5L for COVID-19. Retrospective collection of pre-COVID-19 EQ-5D-5L makes it possible to directly evaluate the impact of COVID-19 on health-related quality of life.

### Electronic supplementary material

Below is the link to the electronic supplementary material.

Supplementary Material 1

## Data Availability

Aggregated data that support the findings of this study are available upon reasonable request from the corresponding author XS, subject to review. These data are not publicly available due to research participant privacy/consent considerations.

## List of Abbreviations

ANOVA: Analysis of variance
ES: Effect size
HRQoL: Health related quality of life
MAIC: Matching-adjusted indirect comparison
SD: Standard deviation
UI: Utility index
EQ VAS: EuroQol visual analogue scale
