# Supplementary material for: Assessment of retrospective collection of EQ-5D-5L in a US COVID-19 population
Source: Health Qual Life Outcomes. 2023 Sep 8;21:103. doi: 10.1186/s12955-023-02187-x (PMC10486034; doi:10.1186/s12955-023-02187-x)
Supplement: Supplementary file 1 — Supplementary Material 1 [file 12955_2023_2187_MOESM1_ESM.docx]

Supplemental Table **1** Patient Characteristics and Retrospective Collection of EQ-5D-5L Utility Index (US Preference Weights) and EQ VAS

|  | Pre-COVID-19 UI | | Pre-COVID-19 VAS | |
| --- | --- | --- | --- | --- |
|  | N (%) | Mean (SD), Median | N (%) | Mean (SD), Median |
| **Total** | 676 | 0.924 (0.117), 0.943) | 674 | 87.4 (10.9), 90.0) |
| **Order of response** |  |  |  |  |
| Standard version first | 353 (52.2) | 0.910 (0.122), 0.943) | 353 (52.4) | 85.3 (11.5), 88.0) |
| Retrospective version first | 323 (47.8) | 0.940 (0.108), 1) | 321 (47.6) | 89.7 (9.8), 92.0) |
| **Age, years** |  |  |  |  |
| Mean, SD | 43.2 (14.7) |  |  |  |
| <25 | 67 (9.9) | 0.900 (0.116), 0.943) | 67 (9.9) | 88.4 (9.3), 90.0) |
| 25-34 | 192 (28.4) | 0.923 (0.104), 0.943) | 192 (28.5) | 87.9 (9.3), 90.0) |
| 35-44 | 153 (22.6) | 0.936 (0.118), 1.000) | 153 (22.7) | 87.8 (10.6), 90.0) |
| 45-54 | 106 (15.7) | 0.910 (0.148), 0.943) | 106 (15.7) | 86.2 (12.6), 90.0) |
| 55-64 | 96 (14.2) | 0.933 (0.107), 1.000) | 95 (14.1) | 85.0 (14.0), 90.0) |
| 65-74 | 57 (8.4) | 0.936 (0.104), 0.943) | 56 (8.3) | 89.1 (9.2), 92.0) |
| ≥75 | 5 (0.7) | 0.944 (0.097), 1.000) | 5 (0.7) | 87.6 (9.0), 84.0) |
| **Gender** |  |  |  |  |
| Female | 495 (73.2) | 0.920 (0.113), 0.943) | 495 (73.4) | 87.2 (11.1), 90.0) |
| Male | 181 (26.8) | 0.935 (0.125), 1) | 179 (26.6) | 87.9 (10.3), 90.0) |
| **Race / Ethnicity** |  |  |  |  |
| Asian | 35 (5.2) | 0.969 (0.051), 1) | 35 (5.2) | 89.9 (9.0), 92.0) |
| Black or African American | 32 (4.7) | 0.918 (0.166), 1) | 32 (4.8) | 82.7 (14.8), 86.5) |
| Hispanic | 85 (12.6) | 0.930 (0.105), 0.943) | 85 (12.6) | 88.4 (11.4), 91.0) |
| Other | 22 (3.3) | 0.899 (0.131), 0.972) | 22 (3.3) | 91.7 (9.1), 93.0) |
| Patient Refused | 16 (2.4) | 0.927 (0.096), 0.943) | 16 (2.4) | 86.6 (10.2), 90.0) |
| White or Caucasian | 486 (71.9) | 0.921 (0.118), 0.943) | 484 (71.8) | 87.1 (10.7), 90.0) |
| **CMS Geographic Region** |  |  |  |  |
| Region 1: ME, NH, VT, MA, CT, RI | 38 (5.6) | 0.941 (0.102), 1) | 38 (5.6) | 87.7 (9.4), 90.0) |
| Region 2: NY, NJ, PR, VI | 17 (2.5) | 0.876 (0.147), 0.943) | 17 (2.5) | 81.9 (18.5), 89.0) |
| Region 3: PA, DE, MD, DC, WV, VA | 66 (9.8) | 0.935 (0.078), 0.943) | 66 (9.8) | 86.3 (9.6), 89.5) |
| Region 4: KY, TN, NC, SC, GA, MS, AL, FL | 247 (36.5) | 0.921 (0.133), 0.943) | 246 (36.5) | 86.8 (11.3), 90.0) |
| Region 5: MN, WI, IL, MI, IN, OH | 92 (13.6) | 0.906 (0.125), 0.943) | 92 (13.7) | 87.0 (10.4), 90.0) |
| Region 6: NM, OK, AR, TX, LA | 128 (18.9) | 0.932 (0.106), 0.972) | 128 (19.0) | 88.2 (10.5), 90.0) |
| Region 7: NE, IA, KS, MO | 26 (3.9) | 0.942 (0.120), 1) | 26 (3.9) | 89.5 (9.9), 90.0) |
| Region 8 : MT, ND, SD, WY, UT, CO | 1 (0.2) | 1 (.), 1) | 1 (0.2) | 90.0 (.), 90.0) |
| Region 9: CA, NV, AZ, GU | 60 (8.9) | 0.931 (0.083), 0.943) | 59 (8.8) | 89.9 (10.6), 93.0) |
| Region 10: AK, WA, OR, ID | 1 (0.2) | 1 (.), 1) | 1 (0.2) | 95.0 (.), 95.0) |
| **US Geographic Region** |  |  |  |  |
| Northeast | 92 (13.6) | 0.922 (0.107), 0.943) | 92 (13.7) | 86.2 (11.9), 90.0) |
| South | 402 (59.5) | 0.926 (0.121), 0.943) | 401 (59.5) | 87.2 (10.9), 90.0) |
| Midwest | 118 (17.5) | 0.914 (0.124), 0.943) | 118 (17.5) | 87.5 (10.3), 90.0) |
| West | 64 (9.5) | 0.935 (0.082), 0.943) | 63 (9.4) | 90.0 (10.3), 93.0) |
| **Social vulnerability index ^a^** |  |  |  |  |
| Mean (SD) | 0.43 (0.21) |  |  |  |
| Category |  |  |  |  |
| <0.25 | 154 (22.8) | 0.941 (0.091), 1) | 154 (22.9) | 88.4 (9.0), 90.0) |
| ≥0.25 and <0.5 | 270 (39.9) | 0.927 (0.112), 0.943) | 268 (39.8) | 87.5 (10.5), 90.0) |
| ≥0.5 and <0.75 | 188 (27.8) | 0.915 (0.124), 0.943) | 188 (27.9) | 86.2 (12.9), 90.0) |
| ≥0.75 | 64 (9.5) | 0.898 (0.156), 0.943) | 64 (9.5) | 87.9 (10.7), 90.0) |
| Previously Tested Positive | 244 (36.1) | 0.924 (0.118), 0.943) | 244 (36.2) | 86.8 (10.9), 90.0) |
| Work in healthcare | 77 (11.4) | 0.938 (0.085), 0.943) | 77 (11.4) | 88.3 (8.7), 90.0) |
| Work in high-risk setting | 71 (10.5) | 0.925 (0.132), 0.943) | 70 (10.4) | 88.6 (10.8), 90.0) |
| Live in high-risk setting | 37 (5.5) | 0.944 (0.091), 1) | 36 (5.3) | 91.2 (9.7), 94.5) |
| **Self-Reported Comorbidity** |  |  |  |  |
| Asthma or Chronic Lung Disease | 58 (8.6) | 0.873 (0.152), 0.940) | 57 (8.5) | 81.7 (14.0), 81.0) |
| Cirrhosis of the liver | 2 (0.3) | 0.581 (0.013), 0.581) | 2 (0.3) | 56.0 (8.5), 56.0) |
| Immunocompromised Conditions or Weakened Immune System **^b^** | 27 (4.0) | 0.889 (0.134), 0.940) | 27 (4.0) | 78.6 (15.2), 80.0) |
| Diabetes | 32 (4.7) | 0.887 (0.131), 0.932) | 32 (4.8) | 82.8 (11.1), 80.5) |
| Heart Conditions or Hypertension | 82 (12.1) | 0.884 (0.158), 0.943) | 80 (11.9) | 82.9 (13.4), 85.0) |
| Overweight or obesity | 27 (4.0) | 0.804 (0.244), 0.883) | 27 (4.0) | 77.8 (13.4), 80.0) |
| At least 1 comorbidity |  |  |  |  |
| **Index day** **^c^ acute COVID-19 symptoms** |  |  |  |  |
| Number of symptoms, Mean (SD) | 5.16 (2.5) |  |  |  |
| Systemic symptoms |  |  |  |  |
| Fever | 236 (34.9) | 0.926 (0.115), 0.943) | 236 (35.0) | 87.7 (11.2), 90.0) |
| Chills | 313 (46.3) | 0.920 (0.124), 0.943) | 313 (46.4) | 87.8 (10.1), 90.0) |
| Muscle or Body Aches | 353 (52.2) | 0.919 (0.123), 0.943) | 353 (52.4) | 86.9 (11.5), 90.0) |
| Headache | 441 (65.2) | 0.919 (0.120), 0.943) | 440 (65.3) | 86.8 (11.2), 90.0) |
| Fatigue | 392 (58.0) | 0.910 (0.127), 0.943) | 391 (58.0) | 86.2 (11.5), 90.0) |
| Respiratory symptoms |  |  |  |  |
| Shortness of Breath or Difficulty Breathing | 87 (12.9) | 0.875 (0.155), 0.940) | 87 (12.9) | 83.3 (12.6), 84.0) |
| Cough | 497 (73.5) | 0.920 (0.118), 0.943) | 497 (73.7) | 87.1 (11.1), 90.0) |
| Sore Throat | 395 (58.4) | 0.927 (0.111), 0.943) | 393 (58.3) | 87.3 (10.9), 90.0) |
| New/Recent Loss of Taste or Smell | 62 (9.2) | 0.877 (0.137), 0.942) | 62 (9.2) | 82.9 (13.3), 85.5) |
| Congestion or Runny Nose | 507 (75.0) | 0.916 (0.122), 0.943) | 506 (75.1) | 86.6 (11.3), 90.0) |
| GI symptoms |  |  |  |  |
| Nausea or Vomiting | 82 (12.1) | 0.904 (0.111), 0.943) | 82 (12.2) | 86.4 (9.3), 89.0) |
| Diarrhea | 120 (17.8) | 0.905 (0.151), 0.943) | 120 (17.8) | 85.7 (12.1), 90.0) |

^a^ CDC/ATSDR Social Vulnerability Index, <https://www.atsdr.cdc.gov/placeandhealth/svi/index.html>

^b^ Immunocompromised conditions includes compromised immune system (such as from immuno-compromising drugs, solid organ or blood stem cell transplant, HIV, or other conditions), conditions that result in a weakened immune system, including cancer treatment, and kidney failure or end stage renal disease

^c^ COVID-19 test nasal swab day

Supplemental Table **2.** Summary of EQ-VAS by EQ-5D-5L Domain Level: Retrospective Collection for Pre-COVID-19 and Standard Collection for COVID-19

|  | Pre-COVID-19 | | | | COVID-19 | | |
| --- | --- | --- | --- | --- | --- | --- | --- |
|  | n (%) | Mean (SD) | Duncan Grouping ^a^ | | n (%) | Mean (SD) | Duncan Grouping ^a^ |
| All | 674 | 87.4 (10.9) |  |  | 671 | 73.3 (16.9) |  |
| Mobility |  |  |  |  |  |  |  |
| No problem | 626 (92.9) | 88.5 (9.5) | A |  | 558 (83.2) | 76.6 (14.3) | A |
| Slight | 40 (5.9) | 75.9 (13.6) | B |  | 88 (13.1) | 60.0 (19.3) | B |
| Moderate | 6 (0.9) | 57.7 (15.8) | C |  | 21 (3.1) | 48.0 (15.4) | C |
| Severe | 2 (0.3) | 50.0 (28.3) | C |  | 4 (0.6) | 39.0 (20.7) | C |
| Unable | (0.0) | 0.0 (0.0) |  |  | (0.0) | 0.0 (0.0) |  |
| Self Care |  |  |  |  |  |  |  |
| No problem | 656 (97.3) | 87.8 (10.3) | A |  | 596 (88.8) | 75.4 (15.3) | A |
| Slight | 17 (2.5) | 70.4 (16.8) | B |  | 60 (8.9) | 61.8 (17.0) | A |
| Moderate | 1 (0.1) | 62.0 (.) | B |  | 13 (1.9) | 40.6 (16.4) | B |
| Severe | (0.0) | 0.0 (0.0) |  |  | 2 (0.3) | 16.5 (16.3) | C |
| Unable | (0.0) | 0.0 (0.0) |  |  | (0.0) | 0.0 (0.0) |  |
| Usual Activity |  |  |  |  |  |  |  |
| No problem | 621 (92.1) | 88.6 (9.6) | A |  | 329 (49.0) | 82.3 (11.9) | A |
| Slight | 36 (5.3) | 78.8 (12.8) | A |  | 207 (30.8) | 71.7 (13.0) | B |
| Moderate | 15 (2.2) | 62.5 (14.4) | B |  | 102 (15.2) | 56.7 (14.5) | C |
| Severe | 2 (0.3) | 57.5 (10.6) | B |  | 24 (3.6) | 45.9 (12.6) | D |
| Unable | (0.0) | 0.0 (0.0) |  |  | 9 (1.3) | 43.7 (23.3) | D |
| Pain / Discomfort |  |  |  |  |  |  |  |
| No problem | 487 (72.3) | 90.1 (8.5) | A |  | 221 (32.9) | 84.2 (11.4) | A |
| Slightly | 146 (21.7) | 83.3 (10.5) | A |  | 307 (45.8) | 73.6 (12.9) | B |
| Moderately | 37 (5.5) | 69.7 (14.9) | B |  | 120 (17.9) | 58.6 (15.6) | C |
| Severely | 4 (0.6) | 62.8 (19.6) | B |  | 20 (3.0) | 46.0 (19.4) | D |
| Extremely | (0.0) | 0.0 (0.0) |  |  | 3 (0.4) | 21.0 (13.9) | E |
| Anxiety / Depression |  |  |  |  |  |  |  |
| No problem | 381 (56.5) | 90.4 (9.0) | A |  | 312 (46.5) | 80.1 (13.6) | A |
| Slightly | 201 (29.8) | 85.5 (10.8) | A | B | 204 (30.4) | 71.8 (15.3) | B |
| Moderately | 73 (10.8) | 78.6 (13.3) |  | B | 113 (16.8) | 64.3 (16.6) | B |
| Severely | 16 (2.4) | 80.3 (11.5) |  | B | 35 (5.2) | 56.0 (18.6) | C |
| Extremely | 3 (0.4) | 78.3 (8.3) |  | B | 7 (1.0) | 46.3 (28.9) | D |

Means of EQ-VAS are significantly difference between levels with different letters (P<0.05) according to Duncan’s new multiple range tests.

Supplemental Table 3. Summary of EQ-VAS by Health States with Volume >10: Retrospective Collection for Pre-COVID-19 and Standard Collection for COVID-19

| Pre-COVID-19 | | | COVID-19 | | | |
| --- | --- | --- | --- | --- | --- | --- |
| Health State | n | EQ-VAS  Mean (SD) | Health State | n | EQ-VAS  Mean (SD) | ARI Symptoms,  Mean (SD) |
| 11111 | 316 | 91.8 (7.6) | 11111 | 130 | 87.8 (9.8) | 4.0 (2.0) |
| 11112 | 124 | 88.2 (9.3) | 11121 | 61 | 80.5 (9.9) | 4.7 (2.3) |
| 11122 | 45 | 84.5 (9.5) | 11221 | 56 | 74.3 (11.5) | 5.6 (2.2) |
| 11121 | 35 | 86.5 (11.4) | 11122 | 43 | 79.1 (9.8) | 5.3 (2.4) |
| 11113 | 30 | 84.3 (7.6) | 11112 | 37 | 83.7 (10.3) | 4.1 (2.4) |
| 11123 | 20 | 82.5 (7.7) | 11222 | 35 | 73.0 (9.8) | 5.3 (2.1) |
|  |  |  | 11223 | 21 | 72.7 (10.2) | 5.3 (2.7) |
|  |  |  | 11211 | 16 | 79.7 (9.5) | 5.0 (2.8) |
|  |  |  | 11113 | 12 | 77.9 (8.1) | 4.6 (3.0) |
|  |  |  | 11123 | 11 | 71.8 (15.0) | 4.8 (2.0) |
|  |  |  | 11212 | 11 | 72.3 (18.5) | 4.2 (2.5) |

Supplemental Table 4. Comparing Patient Characteristics Between Study Cohort and Those Not in Study Cohort

|  | Not in Study Cohort | Study Cohort | | P value |
| --- | --- | --- | --- | --- |
|  | N (%) | N (%) | Response, % |  |
| Total | 39,213 | 676 | 1.7% |  |
| Vaccination status |  |  |  | <0.001 |
| Vaccinated | 22,125 (56.4%) | 465 (68.8%) | 2.1% |  |
| Not Vaccinated | 17,087 (43.6%) | 211 (31.2%) | 1.2% |  |
| Age, years |  |  |  |  |
| Mean (SD) | 42.1 (15.5) | 43.2 (14.7) |  | 0.075 |
| 18-29 | 10,095 (25.7%) | 134 (19.8%) | 1.3% | 0.001 |
| 30-49 | 16,707 (42.6%) | 317 (46.9%) | 1.9% |  |
| 50-64 | 8,561 (21.8%) | 156 (23.1%) | 1.8% |  |
| 65-74 | 2,851 (7.3%) | 62 (9.2%) | 2.1% |  |
| 75+ | 999 (2.6%) | 7 (1.0%) | 0.7% |  |
| Gender |  |  |  | <0.001 |
| Female | 21,370 (54.5%) | 495 (73.2%) | 2.3% |  |
| Male | 17,843 (45.5%) | 181 (26.8%) | 1.0% |  |
| Race / Ethnicity |  |  |  | <0.001 |
| Asian | 2,551 (6.5%) | 35 (5.2%) | 1.4% |  |
| Black or African American | 4,163 (10.6%) | 32 (4.7%) | 0.8% |  |
| Hispanic | 6,878 (17.5%) | 85 (12.6%) | 1.2% |  |
| Other | 1,439 (3.7%) | 22 (3.3%) | 1.5% |  |
| Patient Refused | 1,535 (3.9%) | 16 (2.4%) | 1.0% |  |
| White or Caucasian | 22,647 (57.8%) | 486 (71.9%) | 2.1% |  |
| CMS Geographic Region (n, %) |  |  |  | 0.819 |
| Region 1: ME, NH, VT, MA, CT, RI | 1,734 (4.4%) | 38 (5.6%) | 2.1% |  |
| Region 2: NY, NJ, PR, VI | 957 (2.4%) | 17 (2.5%) | 1.7% |  |
| Region 3: PA, DE, MD, DC, WV, VA | 3,730 (9.5%) | 66 (9.8%) | 1.7% |  |
| Region 4: KY, TN, NC, SC, GA, MS, AL, FL | 14,115 (36.0%) | 247 (36.5%) | 1.7% |  |
| Region 5: MN, WI, IL, MI, IN, OH | 5,091 (13.0%) | 92 (13.6%) | 1.8% |  |
| Region 6: NM, OK, AR, TX, LA | 8,228 (21.0%) | 128 (18.9%) | 1.5% |  |
| Region 7: NE, IA, KS, MO | 1,318 (3.4%) | 26 (3.9%) | 1.9% |  |
| Region 8 : MT, ND, SD, WY, UT, CO | 64 (0.2%) | 1 (0.2%) | 1.5% |  |
| Region 9: CA, NV, AZ, GU | 3,951 (10.1%) | 60 (8.9%) | 1.5% |  |
| Region 10: AK, WA, OR, ID | 25 (0.1%) | 1 (0.2%) | 3.8% |  |
| US Geographic Region |  |  |  | 0.257 |
| Northeast | 6,414 (16.4%) | 118 (17.5%) | 1.8% |  |
| South | 4,387 (11.2%) | 92 (13.6%) | 2.1% |  |
| Midwest | 24,306 (62.0%) | 402 (59.5%) | 1.6% |  |
| West | 4,105 (10.5%) | 64 (9.5%) | 1.5% |  |
| Social vulnerability index |  |  |  |  |
| Mean (SD) | 0.46 (0.22) | 0.43 (0.21) |  | 0.001 |
| Category |  |  |  | 0.046 |
| <0.25 | 7,984 (20.4%) | 154 (22.8%) | 1.9% |  |
| ≥0.25 and <0.5 | 14,432 (36.8%) | 270 (39.9%) | 1.8% |  |
| ≥0.5 and <0.75 | 12,411 (31.6%) | 188 (27.8%) | 1.5% |  |
| ≥0.75 | 4,297 (11.0%) | 64 (9.5%) | 1.5% |  |
| Previously Tested Positive | 14,382 (36.7%) | 244 (36.1%) | 1.7% | 0.756 |
| Work in healthcare | 3,600 (9.2%) | 77 (11.4%) | 2.1% | 0.049 |
| Work in high-risk setting | 4,913 (12.5%) | 71 (10.5%) | 1.4% | 0.114 |
| Live in high-risk setting | 3,021 (7.7%) | 37 (5.5%) | 1.2% | 0.031 |
| Self-Reported Comorbidity |  |  |  |  |
| Asthma or Chronic Lung Disease | 2,412 (6.2%) | 58 (8.6%) | 2.3% | 0.009 |
| Cirrhosis of the liver | 67 (0.2%) | 2 (0.3%) | 2.9% | 0.438 |
| Immunocompromised Conditions or Weakened Immune System b | 1,254 (3.2%) | 27 (4.0%) | 2.1% | 0.244 |
| Diabetes | 2,334 (6.0%) | 32 (4.7%) | 1.4% | 0.184 |
| Heart Conditions or Hypertension | 4,100 (10.5%) | 82 (12.1%) | 2.0% | 0.159 |
| Overweight or obesity | 794 (2.0%) | 27 (4.0%) | 3.3% | <0.001 |
| At least 1 comorbidity | 7,676 (19.6%) | 164 (24.3%) | 2.1% | 0.002 |
| Index day ^c^ acute COVID-19 symptoms |  |  |  |  |
| Number of symptoms, Mean (SD) | 5.1 (2.5) | 5.2 (2.5) |  | 0.731 |
| Systemic symptoms |  |  |  |  |
| Fever | 15,502 (39.5%) | 236 (34.9%) | 1.5% | 0.195 |
| Chills | 19,143 (48.8%) | 313 (46.3%) | 1.6% | 0.824 |
| Muscle or Body Aches | 20,646 (52.6%) | 353 (52.2%) | 1.7% | 0.615 |
| Headache | 25,215 (64.3%) | 441 (65.2%) | 1.7% | 0.197 |
| Fatigue | 21,765 (55.5%) | 392 (58.0%) | 1.8% | 0.522 |
| Respiratory symptoms |  |  |  |  |
| Shortness of Breath or Difficulty Breathing | 5,382 (13.7%) | 87 (12.9%) | 1.6% |  |
| Cough | 29,232 (74.5%) | 497 (73.5%) | 1.7% | 0.545 |
| Sore Throat | 20,619 (52.6%) | 395 (58.4%) | 1.9% | 0.003 |
| New/Recent Loss of Taste or Smell | 3,782 (9.6%) | 62 (9.2%) | 1.6% | 0.680 |
| Congestion or Runny Nose | 27,240 (69.5%) | 507 (75.0%) | 1.8% | 0.002 |
| GI symptoms |  |  |  |  |
| Nausea or Vomiting | 5,559 (14.2%) | 82 (12.1%) | 1.5% | 0.130 |
| Diarrhea | 6,752 (17.2%) | 120 (17.8%) | 1.7% | 0.716 |
